# Periodic Organic–Inorganic Halide Perovskite Microplatelet Arrays on Silicon Substrates for Room‐Temperature Lasing

**DOI:** 10.1002/advs.201600137

**Published:** 2016-05-30

**Authors:** Xinfeng Liu, Lin Niu, Chunyang Wu, Chunxiao Cong, Hong Wang, Qingsheng Zeng, Haiyong He, Qundong Fu, Wei Fu, Ting Yu, Chuanhong Jin, Zheng Liu, Tze Chien Sum

**Affiliations:** ^1^ CAS Center for Excellence in Nanoscience and CAS Key Laboratory of Standardization and Measurement for NanotechnologyNational Center for Nanoscience and TechnologyBeijing100190P.R. China; ^2^ Division of Physics and Applied PhysicsSchool of Physical and Mathematical SciencesNanyang Technological UniversitySingapore637371Singapore; ^3^ Center for Programmable MaterialsSchool of Materials Science & EngineeringNanyang Technological UniversitySingapore639798Singapore; ^4^ State Key Laboratory of Silicon MaterialsSchool of Materials Science and EngineeringZhejiang UniversityHangzhou310027P.R. China; ^5^ NOVITASNanoelectronics Centre of ExcellenceSchool of Electrical and Electronic EngineeringNanyang Technological UniversitySingapore639798Singapore; ^6^ Energy Research Institute @ NTU (ERI@N)Nanyang Technological University50 Nanyang DriveSingapore637553Singapore

**Keywords:** array, BN, lead halide perovskite, single mode laser, whispering‐gallery‐mode

## Abstract

Organic–inorganic metal halide perovskites have recently demonstrated outstanding efficiencies in photovoltaics as well as highly promising performances for a wide range of optoelectronic applications such as lasing, light emission, optical detectors, and even for radiation detection. Key to the realization of functional perovskite micro/nanosystems on the ubiquitous silicon optoelectronics platform is through sophisticated lithography. Despite the rapid progress made in halide perovskite lasing, direct lithographic patterning of perovskite films to form optical cavities on conventional substrates remains extremely challenging. This study realizes room‐temperature high‐quality factor whispering‐gallery‐mode lasing (*Q* ≈ 1210) from patterned lead halide perovskite microplatelets fabricated in periodic arrays on silicon substrate with micropatterned BN film as the buffer layer. By varying the size of the platelets, modal selectivity for single mode lasing can be achieved with different cavity sizes or by simply breaking the structural symmetry of the cavity through designing the pattern. Importantly, this work demonstrates a straightforward, versatile bottom‐up scalable strategy to realize high‐quality periodic perovskite arrays with variable cavity sizes for large‐area light‐emitting and optical gain applications.

## 1 Introduction

Organic–inorganic halide perovskites (CH_3_NH_3_PbX_3_ where X = Cl, Br, I) have recently emerged as one of the most promising materials for photovoltaics with certified power conversion efficiencies exceeding 20.1%.^1^, ^2^, ^3^, ^4^, ^5^, ^6^, ^7^, ^8^ This outstanding performance can be traced to their long carrier lifetimes, long diffusion length, and low trap densities.^9^, ^10^, ^11^, ^12^ These excellent properties, along with their high quantum yield and wavelength tunability, make halide perovskites ideal for light‐emission applications such as for light‐emitting diodes (LEDs), light‐emitting field‐effect transistors, and lasing.^13^, ^14^, ^15^, ^16^, ^17^, ^18^, ^19^ Since the first demonstration of ultrastable amplified spontaneous emission (ASE) with broad spectral tunability from perovskite thin films and lasing from perovskite crystals,^20^ perovskite lasing in a broad range of cavity structures, including microplatelets,^21^ nanowires,^22^ microspheres,^23^ and distributed Bragg reflection (DBR) mirrors,^24^ have been realized.^25^ Notably, Zhu et al. recently reported a solution‐processed technique to synthesize single‐crystal perovskite nanowires and demonstrated their application on lasers with high‐quality factor and low lasing threshold.^26^ However, few works focus on the following two highly important features: (i) finding a straightforward means for selective or directed and/or periodic placement of perovskite micro/nanostructures; and (ii) the compatibility of substrates on which perovskite structures were grown for further optoelectronic integration.^27^, ^28^ Consequently, controlled growth or pattering of these perovskite building blocks on the ubiquitous silicon optoelectronics platform is a crucial step toward realization of practical optoelectronic devices. However, the vulnerability of the organic matrix in organic–inorganic halide perovskites makes it incompatible and impractical for direct lithographic patterning of perovskite films with beam techniques and with solution‐based or dry etching approaches that would severely degrade the quality and optical properties of the perovskite structure. This limitation is especially debilitating for optical gain applications where cavity quality is of paramount importance. In ref. 27, the authors had proposed one method based on assembled monolayers of hydrophobic OTS to fabricate patterned perovskite arrays and they studied the electronic properties of as‐prepared perovskite crystals. In contrast, herein, we use the single layer material as a buffer layer to synthesize the pattered perovskites. We then systematically studied the optical properties of these pattered crystals, which are extremely important for diverse optoelectronic systems such as photodetectors, LEDs, and laser diodes.

In this work, we utilized a novel bottom‐up growth technology for the synthesis high quality patterned perovskite arrays on Si with varying cavity sizes for light emission and lasing, enabled by pre‐patterned single layer hexagonal boron nitride (h‐BN) buffer layer. The atomically thin crystalline, single layer h‐BN materials^29^, ^30^, ^31^ function as the intermediate layer and growth nuclei that provide not only an epitaxial intermediary between the substrate and the perovskite, but also as a cladding layer for better optical confinement in lasing due to its large bandgap. Furthermore, the h‐BN can also serve as a superior insulating dielectric layer (as compared to other layered materials like graphene, MoS_2_ and black phosphor) for preventing shorts in practical devices.^28^, ^32^, ^33^ Most essentially, the superior optical properties of the perovskite structure itself demanded by lasing applications are not be drastically compromised with this patterning approach. Based on this technology, optically pumped room temperature whispering‐gallery‐mode (WGM) lasing with *Q* factors up to ≈1210 from individual perovskite platelets was obtained. Furthermore, mode selection, which is an important issue for the application of such microstructured coherent light sources, was also demonstrated. By changing the size of cavity or by breaking the symmetry of the cavity with the patterning approaches, wavelength tunable, single mode lasers were realized. This patterning approach provides greater versatility over the control of the lasing mode and its wavelength through judicious design of the perovskite cavity. Our work suggest a viable, scalable lithography approach to fabricating high‐quality periodic light emitting arrays for potential applications as integrated coherent light sources and other large‐area optoelectronic applications.^34^, ^35^, ^36^

## 2 Results and Discussion

**Figure 1:**
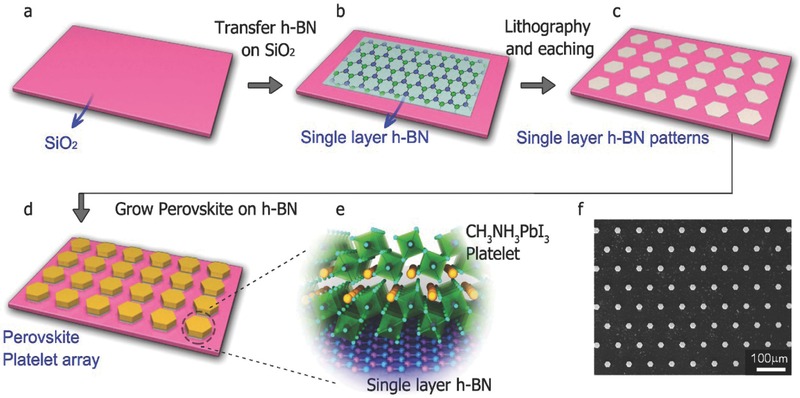
Fabrication process for the perovskite platelet array. a–e) Schematic illustration of fabrication process used to grow hexagonal lead halide perovskite (CH_3_NH_3_PbX_3_, X = Cl, Br, I, here I was used here as representative example) microplatelet arrays on BN (Boron‐Nitride) patterned films. f) SEM image of the as‐prepared CH_3_NH_3_PbX_3_ platelet array. The edge length of hexagonal platelet is ≈15 μm and the gap distance between all adjacent platelets is ≈50 μm.

**Figure** **1**a–e presents a schematic illustration of the fabrication processes that is used to grow hexagonal lead halide perovskite microplatelet arrays on pre‐patterned h‐BN films. BN films, which are first synthesized on Cu foil by chemical vapor deposition (CVD), were transferred onto SiO_2_/Si substrates using a standard transfer method. After that, the large‐area BN film (wafer scale) was patterned using standard photo‐lithography methods. The epitaxial growth of highly crystalline PbI_2_ platelets on these BN substrates are performed via a physical vapor deposition (PVD) process. The PbI_2_ crystals were then converted into CH_3_NH_3_PbI_3_ by reacting with CH_3_NH_3_I under vacuum. There are two key points in the growth of perovskite/BN vdW solids: nucleation site and van der Waals epitaxy. PbI_2_ nanoparticles first nucleated around the edges and defects of 2D materials. These nanoparticles then grew along surface of 2D materials and began to merge to form highly crystalline nanoplatelets. Lastly, the PbI_2_ nanoplatelets extended on BN material surface and covered all the area. Figure 1f shows the scanning electron microscope (SEM) images of highly periodic perovskite microplatelet arrays with comparable edge size and thickness. The edge length of these samples is around ≈15 μm and thickness of ≈200 nm (Figure S1, Supporting Information). By shape designing of BN pattern and then controlling the growth time, perovskite arrays with variable shapes and thicknesses can also be fabricated (for example, nanowire arrays, Figure S2, Supporting Information).

**Figure 2:**
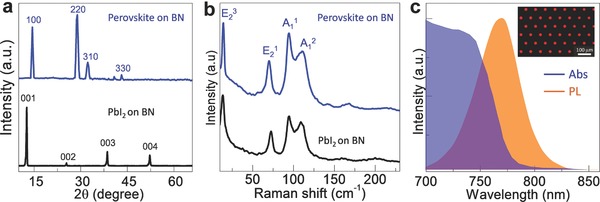
Characterization of PbI_2_ and converted perovskite platelet array. a) X‐ray diffraction (XRD) patterns of as‐grown PbI_2_ and perovskite on BN. The set of strong peaks at 12.54°, 25.34°, 38.50°, and 52.24°, assigned to 001, 002, 003, and 004 of the PbI_2_ crystal growing on CVD BN pattern—indicating a high level of phase purity in the PbI_2_ hexagonal crystal structure whose highly oriented growth direction is along the c‐axis. After conversion, the characteristic peaks at 14.06°, 28.38°, 3l.74°, and 43.14°, are assigned to (110), (220), (310), and (330) for CH_3_NH_3_PbI_3_ perovskite with a tetragonal crystal structure. b) The corresponding Raman spectra of samples before and after conversion. For both the PbI_2_ and perovskite platelets, Raman spectra show peaks at 14, 70, 94, and 110 cm^−1^ that are assigned to *E*_2_^3^, *E*_2_^1^, *A*_1_^1^, and *A*_1_^2^, respectively. c) Absorption (blue) and photoluminescence (orange) spectra taken from perovskite platelets at room temperature. The energy gap of CH_3_NH_3_PbI_3_ is around 1.60 eV (corresponding 780 nm). Inset is the PL image of patterned perovskite arrays at the mapping wavelength of 780 nm.

**Figure 3:**
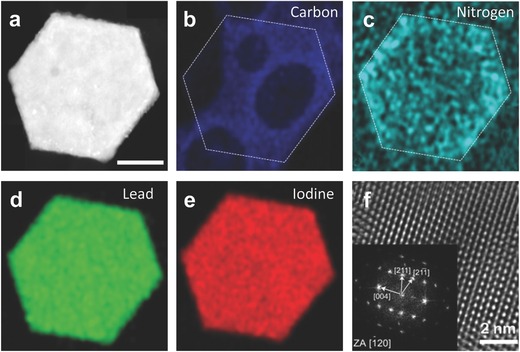
Transmission electron microscope (TEM) characterization of the perovskite/BN platelet. a) Low‐magnification STEM image of a representative perovskite platelet. The scale bar is 5 μm. b–e) Element (C, N, Pb and I) mapping obtained by energy‐dispersive X‐ray spectroscopy shows the uniformity of the elemental distribution in the perovskite platelet. f) High‐resolution TEM (HRTEM) image showing the structure of the perovskite platelet. Inset is the corresponding fast Fourier transform pattern from this HRTEM image along the [‐102] zone axis (ZA).

In order to validate the complete conversion of PbI_2_ to CH_3_NH_3_PbI_3_, we first investigate the X‐ray diffraction (XRD) patterns of the arrays before and after conversion. Before conversion, a set of strong peaks at 12.54°, 25.34°, 38.50°, and 52.24° (**Figure** **2**a),^37^ which are assigned to (001), (002), (003), and (004) of the PbI_2_ crystal grown on CVD BN, respectively. This indicates a high level of phase purity for the hexagonal crystal structure of PbI_2_ which has a highly oriented growth direction along the c‐axis. After conversion, CH_3_NH_3_PbI_3_ with a tetragonal crystal structure were formed – evident from the characteristic peaks at 14.06°, 28.38°, 31.74°, and 43.14°, which are assigned to (110), (220), (310), and (330) for pure CH_3_NH_3_PbI_3_ crystals, respectively.^28^ From Raman measurements, PbI_2_ and CH_3_NH_3_PbI_3_ possess almost the same Raman spectra (Figure 2b) indicating that the perovskite platelet retains the 4H polytype of PbI_2_.^38^ The phonon vibration peaks at 14 cm^−1^ is assigned to *E_2_^3^*, the shear‐motion rigid‐layer mode of PbI_2_ with 4H polytype; while the peaks at ≈70 cm^−1^, ≈94 cm^−1^ and ≈110 cm^−1^ are assigned to *E_2_^1^*, *A_1_^1^*, and *A_1_^2^*, respectively.^39^ Figure 2c shows the absorption (Abs) and photoluminescence (PL) spectra measured for perovskite platelets at room temperature. The energy gap extracted from the absorbance spectra (the optical band gap was deduced from the slope of the absorption edge) is around 780 nm, which is in good agreement with our PL data and from previous reports.^40^ The absence of emission peak at around 520 nm (not shown here) suggests the minimal presence or absence of PbI_2_ – thus attesting the optical purity of as‐prepared perovskite platelet. Inset is the PL image of patterned perovskite arrays at the mapping wavelength of ≈780 nm. The quality of as‐grown perovskite platelet samples was further examined with selected area electron diffraction (SAED), high‐resolution transmission electron microscope (HRTEM) imaging and energy dispersive X‐ray spectroscopy (EDX). **Figure** **3**a shows a typical perovskite flake and the corresponding elemental mapping of carbon (C), nitrogen (N), lead (Pb) and iodine (I) are presented in Figure 3b–e. These mapping images confirm the elemental uniformity of the perovskite over the whole area after conversion—as compared to their spin‐coated polycrystalline perovskite counterparts. In addition to the XRD spectra, the crystal structure of the converted CH_3_NH_3_PbI_3_ is evaluated by HRTEM imaging, as shown in Figure 3f. The perovskite lattice is quite clear and inset shows the corresponding fast Fourier Transform pattern from this HRTEM image along the [−120] zone axis (ZA).

**Figure 4:**
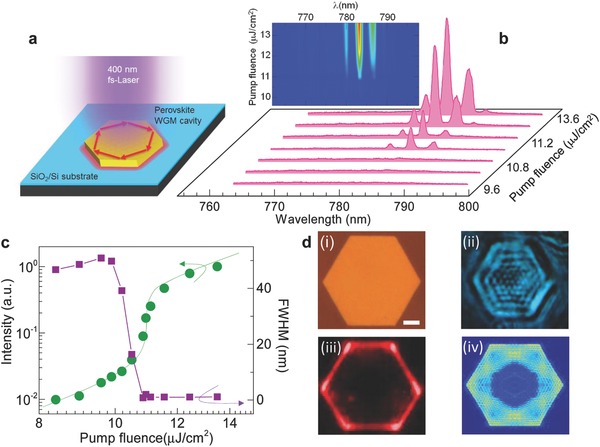
Lasing characterizations from a representative CH_3_NH_3_PbI_3_ platelet. a) Schematic of CH_3_NH_3_PbI_3_ platelet on silicon substrate pumped by 400 nm laser excitation (≈50 fs, 1 kHz). b) Emission spectra at different pump fluences recorded at around the lasing threshold. Inset is the 2D pseudo‐color plot of emission spectra under different pump fluences (*P*). A broad SPE (spontaneous emission) is observed below the threshold (≈10 μJ cm^−2^), and narrow lasing peaks can be seen above the threshold. c) Integrated emission intensity and FWHM (full‐width at half‐maximum) as a function of *P* showing the lasing threshold of ≈11 μJ cm^−2^. The FWHM of lasing peak (∆λ) at ≈12 μJ cm^−2^ is ≈0.64 nm, corresponding to a Q factor ≈1210. d) (i) Optical image of a representative perovskite platelet with edge length (*L* = 15 μm). (ii) The image is the scattering image of 400 nm excitation laser obtained from the imaging system. (iii) The image shows the emission of perovskite above threshold with excitation pump fluence of ≈15 μJ cm^−2^. The excitation wavelength of 400 nm is filtered by a 425 nm long pass filter. Six bright spots are observed at the corner of the platelet, which are attributed to the scattering from the coherent interference under lasing operation. (iv) The image is the simulated field distribution at resonant cavity mode of typical hexagonal perovskite platelet using TM mode. All the images have the same scale bar (i.e., 5 μm).

**Figure** **4** shows the clear evidence of room temperature lasing from perovskite platelets in the array sample. Figure 4a presents a schematic of an individual perovskite platelet on the silicon substrate, which is optically pumped using 400 nm fs pulses. To uniformly excite the platelet, the pump laser was focused to a beam of diameter ≈40 μm to cover the entire platelet. All optical measurements were performed with the samples in vacuum or under inert‐gas. Figure 4b shows the emission spectra from the perovskite platelet at different excitation powers near the threshold, and the left inset shows the normalized spectral map. At low pump fluence (≈8.4 μJ cm^−2^), the emission spectrum is broad and the intensity is low. With increasing pump fluence (≈9.0 μJ cm^−2^), the emission peak broadens due to band filling effect and increases in intensity.^41^ For pump fluence between 9.6 and 10.8 μJ cm^−2^ (per pulse), a series of sharp peaks is observed at the shoulder of the main peak, which is amplified due to the optical feedback in the WGM cavity. At the pump fluence of ≈11 μJ cm^−2^, the intensity of the sharp peaks increase sharply, which is a signature of lasing behavior. Figure 4c presents another clear evidence of lasing – the nonlinear increase of the emission peak intensity as a function of excitation (light input‐light output, or ‘*L–L*' curve) with a characteristic ‘knee’ or ‘kink’ is observed in the *L–L* curve and an abrupt shortening (see Figure S3, Supporting Information) of the full width half maximum (FWHM) of the platelet emission above the threshold pump fluence of ≈11 μJ cm^−2^. The solid line is fitted by using a multi‐mode lasing model described in a previous report (see ref. 42, equation 20),^42^, ^43^ which gives a threshold of ≈11 μJ cm^−2^ and fitting parameter *x*_0_ of ≈0.05, which is related to the gain saturation of individual longitudinal laser modes and their lateral mode area.^43^ A higher value of *x_0_* corresponds to a smaller mode area and a higher *β* factor. Above threshold, the lasing peak intensity increase linearly with excitation, as shown in Figure 4c. From time‐resolved PL measurements, the dominant fast recombination lifetime of around 30 ps (which is limited by the time‐resolution of our streak camera over the scan window—see Supporting Information, Figure S4) is also another signature of the lasing behavior.

Figure 4d (i) is the optical image of perovskite platelet with white light illumination. Clear diffraction patterns of laser source (400 nm) can be seen from the far‐field optical image without white light illumination (Figure 4d (ii)). The patterns located inside the platelet shows familiar hexagonal outlines, indicating good optical confinement within the platelet WGM cavities. The corresponding simulation results (Figure S5, Supporting Information) suggest that the pattern originates from the interference of light inside the cavity. Figure 4d (iii) shows the emission of perovskite above threshold with excitation pump fluence of ≈15 μJ cm^−2^. The excitation wavelength of 400 nm is filtered out with a 425 nm long pass filter. Six bright spots are observed at the corner of the platelet, which are attributed to scattered light from coherent interference under lasing operation. The image shown in Figure 4d (iv) is the simulated field distribution of the resonant cavity mode for a typical hexagonal perovskite platelet when the TM mode dominates. The simulation pattern is very similar to the far‐field image as shown in Figure 4d (iii), suggesting that the TM mode yields a lower threshold due to its larger effective index compared with the TE mode. Our previous work, which had focused on the optical properties of single crystal PbI_2_, has some resemblance in shape and electrical field distribution.^40^ However, PbI_2_ has a hexagonal structure with a lattice constant *c* = 0.695 nm with an orientation perpendicular to the substrate; while after conversion to perovskite, it has a tetragonal structure (lattice *c* = 1.244 nm) with a large expansion in the c axis. Moreover, PbI_2_ has a bandgap of ≈2.4 eV and the bandgap for perovksite is ≈1.6 eV after conversion. The perovskite crystals also show superior optical properties, owing to the lower threshold, higher *Q* factor and near infrared working wavelength range.

**Figure 5:**
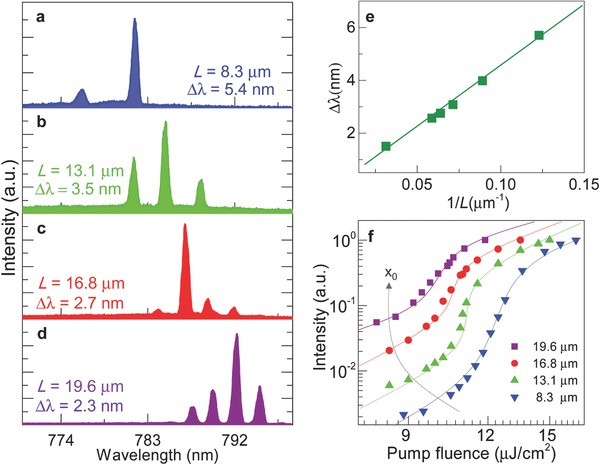
Edge‐length‐dependent lasing behavior for different size perovskite platelets. a–d) Lasing spectra are dependent on the edge length of hexagonal perovskite whispering‐gallery‐mode cavity. The edge lengths are 8.3, 13.1, 16.8, and 19.6 μm (also indicted), respectively, from upper to the bottom. The thickness is ≈180 nm. The lasing wavelength redshifts and mode spacing deceases as a function of the hexagonal cavity edge length. e) Mode spacing of the lasing peaks as a function of the reciprocal edge length (green dots). The experimental data are well‐fitted by a linear function (green line) with intercept at zero. f) Logarithmic plot of the experimental laser output power with increasing pump fluence, showing threshold region as a ‘kink’ between spontaneous emission and lasing. The different color points represent the experimental data of four hexagonal perovskite platelets with different edge length as shown in panels (a)–(d). The corresponding lines represent the fit to experimental data using multimode laser model with the fitting parameter *x_0_* = 0.3 (*L* = 19.6 μm), 0.2 (*L* = 16.8 μm ), 0.08 ( *L* = 13.1 μm ), and 0.02 (*L* = 8.3 μm), respectively.

For the perovskite platelet with a different size, the lasing modes can be tuned due to the intrinsic self‐absorption in the perovskite cavity. We first fabricated perovskite platelet arrays with platelets of a different size using the above mentioned method. As the edge length *L* increases from 8.3, 13.1, 16.8 to 19.6 μm (thickness of ≈200 ± 10 nm), the highest optical gain area where lasing can be realized, redshift to the lower energy region (**Figure** **5**a–d). The strongest lasing modes of the perovskite platelets are extracted from samples with various lengths of edges. It shows an exponential decrease as a function of the edge length according to the self‐absorption equations (Figure S6, Supporting Information). The limit of the lasing modes occurs at ≈792 nm as deduced from the fitting, which is close to the Urbach tail near the bottom of the absorption edge (see Figure 2c), thus further validating the dependence of the self‐absorption in the cavity. Another feature observed in Figure 5a–d is the mode spacing decreases from 5.4 to 2.3 nm when the edge length *L* increases from 8.3 to 19.6 μm. The mode spacing in the WGM oscillation cavity is predicted by the relationship Δ*λ* = *λ* ^2^/6*nL*,^44^ where *L* is the edge length and *n* is effective refractive index at wavelength of *λ*.^45^ Therefore, for a fixed *λ*, the mode spacing (Δ*λ*) should scale proportionally to the inverse length 1/*L*. This behavior is illustrated in Figure 5e. The linear fit between Δ*λ* and 1/*L*, suggests WGM mode lasing in these perovskite platelet cavities.

To estimate the lasing thresholds and spontaneous emission coupling factor (*β*) of an individual platelet laser of different sizes, circularly polarized pump excitations were used for the measurements in the light–light (*L–L*) plots shown in Figure 5f. Similarly, using the multimode lasing model, we fitted the experimental data by using different values of fitting parameter *x*_0_. A higher value of *x*_0_ corresponds to a smaller mode area and a higher *β* factor. With increasing platelet size from 8.3, 13.1, 16.8 to 19.6 μm, the value of *x*_0_ increases from 0.02, 0.08, 0.2 to 0.3; while the corresponding lasing threshold decreases from 12.8, 10.8, 10.0 to 9.0 μJ cm^−2^. It is possible that the size dependent lasing threshold may be due to the different trap densities in different sized platelets, which influences the charge carrier dynamics. Although such mechanism was proposed in some early works,^46^, ^47^ it should be noted that the samples in these two cases are nanoparticles. The surface trap states which are dominant in their recombination process result in large differences in the emission lifetimes of perovskite nanoparticles of different sizes. However, in our work, the size of the perovskite platelet is relatively large (8–20 μm in edge length). Surface trap states is not the predominant factor that affects the recombination processes. Indeed, Figure S7 (Supporting Information) shows that different sized perovskite platelets possess similar decay lifetimes, which suggests that the different platelet size has little influence on the charge carrier dynamics. Hence, this different trap density mechanism could be excluded. Instead, based on the definition of *Q* = *λ*/Δ*λ*, the *Q* factor was estimated to be ≈1200. This suggests that there is more coupling of the spontaneous emission into the lasing modes with lower losses in the bigger samples. This behavior is consistent with previous descriptions that the lasing threshold power density (*P*_thr_) is inversely proportional to the *β* × Q product,^48^, ^49^ where the larger *β* and Q factors would mean a lower threshold.

**Figure 6:**
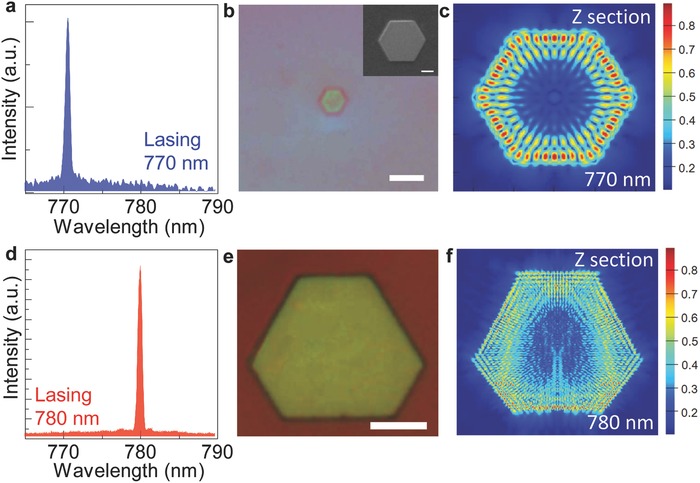
Single mode lasing behavior in small size perovskite platelet and symmetry‐broken platelet. a) Single mode lasing observed in a small hexagonal shaped perovskite platelet. b) The optical image of the corresponding small hexagonal shaped perovskite platelet in panel (a), the scale bar for SEM is 1 μm and for the optical image is 5 μm. c) Simulated field distribution at resonant cavity mode (780 nm) of the small hexagonal perovskite platelet using TM mode. d) Single mode lasing is observed in a nonregular hexagonal perovskite platelet. Panels (e) and (f) are the optical image and simulated field distribution at resonant wavelength of 770 nm by using TM mode. The scale bar is 5 μm.

Mode selectivity is another important feature for potential applications of nano/microlaser sources Typically, the lasing emission of the platelet is usually multimodal in the absence of any mode selectivity mechanism. A general method to obtain single mode lasing is designing and fabricating the multilayered films or gratings that form DBR or distributed feedback structures.^50^, ^51^ However, this method typically requires complicated and time‐consuming fabrication processes (e.g., very stringent high resolution requirements with electron beam lithography). Another possible way is to select a common mode from two coupled cavities by using the Vernier effect.^52^, ^53^ The third approach^54^ is based on expanding the free space range of the multimodes by significantly shortening the optical path of the lasing cavity, until only one mode is left.^55^ As shown in **Figure** **6**a–c, when the platelet decreases to ≈2 μm, only one lasing mode can be observed. From the simulation data (see Figure 6c), we can clearly see the mode distribution in the *x–y* plane of the hexagonal shaped cavity. However, shortening the cavity path will inevitably reduce the round‐trip gain and increase the optical loss, resulting in higher thresholds for lasing action. To solve this problem, an alternative way for single mode lasing can be employed through breaking the symmetry of cavity structure. It will result in modal gain that is selected by the asymmetrical structure of the cavity.^56^ Figure 6d–f demonstrates the realization of single mode lasing inside an asymmetrically shaped cavity. The lasing wavelength located at around 780 nm for the asymmetrically shaped cavity (as shown in Figure 6e) and the threshold is around 12 μJ cm^−2^; while for a 2 μm edge‐length cavity, the lasing wavelength is around 770 nm with a higher threshold of around 37 μJ cm^−2^.

## 3 Conclusions

In summary, we fabricated high‐quality lead halide perovskite microplatelet arrays on silicon substrate by using micropatterned single layer BN film as the buffer layer. The single layer BN, acting as the intermediate layer and growth nuclei, provides not only epitaxial affiliation between the substrate and the perovskite, but also better optical confinement for lasing. The perovskite microplatelet arrays possess both good crystalline and high optical quality. Whispering‐gallery‐mode lasing from the perovskite platelet with *Q* factor as large as 1210 was observed at room temperature. By shortening size of the cavity or through breaking the symmetry of the cavity, single mode lasing has been demonstrated. Our experimental results showcase a straightforward bottom‐up scalable patterning strategy to realize high‐quality periodic perovskite arrays with variable cavity sizes for large‐area light‐emitting and optoelectronic applications.

## 4 Experimental Section

*BN Patterns Fabrication*: As‐transferred BN film was used to prepare various patterns for inducing the growth of PbI_2_ patterns. The fabrication process consisted of the following steps: (1) BN film was covered by a photoresist layer spin‐coated (AZ5214, 4000 *r* min^−1^) on top of the h‐BN surface; (2) standard photolithography was performed to pattern the photoresist layer as a mask; (3) argon‐based plasma etching (power is 50 W, pressure is 200 mTorr, and time is 30 s) was performed to transfer the photoresist mask pattern onto underlying h‐BN; (4) photoresist mask was completely removed in acetone, and a BN pattern was created, such as the hexagonal and wire patterns shown in the main and supporting text.

*Synthesis of PbI_2_ Platelets*: PbI_2_ powder (99.999%, Aldrich) was used as a single source and placed into a quartz tube mounted on a single‐zone furnace (Lindberg/Blue MTF55035C‐1). The BN pattern substrate (1 × 3 cm^2^) was placed in the downstream region inside the quartz tube. The quartz tube was first evacuated to a base pressure of 3 mTorr, followed by a 50 sccm flow of high‐purity Ar gas. The temperature and pressure inside the quartz tube were set and stabilized to desired values for each halide (390 °C and 30 Torr). In all cases, the synthesis was carried out within 30 min and the furnace was allowed to cool down naturally to ambient temperature.

*Synthesis of CH_3_NH_3_I*: The synthesis of CH_3_NH_3_I: 18.0 mL of methylamine (40 wt% in water, Sigma) was dissolved in 100 mL of absolute ethanol (Absolute for analysis, Merck Millipores) in a 250 mL round bottom flask. 20.0 mL of hydroiodic acid (57 wt% in water, Alfa aesar) was added into the solution dropwise. After that, the solution was stirred at 0 °C for 2 h. The raw product was obtained by removing the solvents using a rotary evaporator. A recrystallization process of the raw product, including the re‐dissolution in 80 mL absolute ethanol and the precipitation after the addition of 300 mL diethyl ether, was carried out twice to obtain a much purer product. Finally, the white powders were collected and dried at 60 °C for 24 h in a vacuum oven.

*Synthesis of Perovskites via the Reaction with CH_3_NH_3_I and PbI_2_*: The conversions were done using a similar CVD system. CH_3_NH_3_I were used as a source and placed in the center of the quartz tube while CVD BN substrate having as‐grown lead halide platelets were placed around 5–6 cm away from the center in the downstream region. The quartz tube was first evacuated to a base pressure of 3 mTorr, followed by a 50 sccm flow of high‐purity Ar gas. The pressure was then stabilized to 30 Torr and the temperature was elevated to 120 °C and kept there for 1 h after which the furnace was allowed to cool down naturally to ambient temperature.

*Photoluminescence and Lasing Measurements*: A frequency‐doubled, mode‐locked Ti‐sapphire regenerative amplifier laser (Coherent Inc, Libra) was used to pump the photonic lasers (pump wavelength 400 nm, repetition rate 1 kHz, pulse duration 50 fs). An objective lens (20×, numerical aperture 0.35) was used to focus the pump beam to a 40 μm diameter spot on the sample. All experiments were carried out at room temperature. Individual spectra were recorded using a spectrometer with a resolution of 0.30 nm and an electrically cooled charge coupled device (CCD) (Princeton Instruments).

*Time‐Resolved Photoluminescence Measurement*: The lifetime measurements were conducted under very low pump conditions to avoid sample heating and multiparticle scattering effects using a streak camera (Optronics GmbH); the laser source used here was a frequency‐doubled mode‐lock Ti‐sapphire oscillator laser (pump wavelength 400 nm, repetition rate 76 MHz, pulse length 120 fs). A 425 nm long pass filter was used to filter out laser source from the PL emission.

*XRD, SEM, AFM, TEM, PL, and Raman Measurements*: X‐ray diffraction pattern (2*θ* scans) were obtained from perovskite platelet supported on the SiO_2_/Si substrates using an X‐ray diffractometer (XRD Shimadzu Thin Film), using Cu‐Kα radiation (*λ* = 1.54050 Å) within a diffraction angle (2*θ*) from 5° to 60°. The SEM images of perovskite platelet were obtained using JEOL JEM7600F operated at an accelerating voltage of 10 kV. For all the AFM, experiments were performed in tapping mode under ambient conditions (Dimension ICON SPM system, Bruker, USA). Commercial silicon tips with a nominal spring constant of 40 N m^−1^ and resonant frequency of 300 kHz were used in all the experiments. The PbI_2_ samples for TEM were flaked off from the 2D substrates by using Toluene (99.85%, Acros Organics) and then transfer onto the TEM grids (Quantifoil Mo grids). The CH_3_NH_3_PbI_3_ perovskite samples for TEM measurement were converted from the PbI_2_ onto the TEM grids, with the similar method introduced in the materials synthesis part above. The HRTEM and the SAED pattern were done with an FEI Tecnai F20 operated with an acceleration voltage of 200 kV. The chemical composition of lead iodide and CH_3_NH_3_PbI_3_ perovskite was determined by means of EDX (attached to FEI Tecnai F20). A WITec alpha300 RAS Raman system with a piezocrystal controlled scanning stage, an objective lens of 100× magnification (numerical aperture, NA = 0.95), and an electron multiplying CCD was used for recording PL and Raman spectra/images. For PL spectra/images, a 600 lines mm^−1^ grating was used. For Raman spectra/images, a low‐wavenumber coupler and a 2400 lines mm^−1^ grating were used for observing low‐frequency Raman modes and achieving a good spectral resolution. All the PL and Raman spectra/images were recorded under an excitation laser of 532 nm (*E*_laser_ = 2.33 eV). To avoid the laser‐induced heating, laser power was kept below 0.1 mW. The laser spot was of ≈0.5 μm in diameter. The fluorescence image was obtained by an Olympus fluorescence microscope under green excitation of 530–550 nm with a mercury lamp as the excitation light source. UV–vis absorption spectra of perovskite prepared on quartz were recorded on SHIMADZU UV‐3101PC UV–vis–NIR scanning spectrophotometer.

*Simulation Details*: Simulations of lasing mode distributions were first performed in 1D (*z‐*direction) using the eigenmode method (MODE Solutions from Lumerical Solutions), and the simulated effective mode index was used to obtain the 2D (*x* and *y* directions) mode distributions using the finite‐difference time‐domain (FDTD) method (FDTD Solutions from Lumerical Solutions). The refractive index of perovskite was obtained from previous references.^20^, ^21^

## Supporting information

As a service to our authors and readers, this journal provides supporting information supplied by the authors. Such materials are peer reviewed and may be re‐organized for online delivery, but are not copy‐edited or typeset. Technical support issues arising from supporting information (other than missing files) should be addressed to the authors.

Supplementary
