# Supplementary material for: Periodic Organic–Inorganic Halide Perovskite Microplatelet Arrays on Silicon Substrates for Room‐Temperature Lasing
Source: Adv Sci (Weinh). 2016 May 30;3(11):1600137. doi: 10.1002/advs.201600137 (PMC5102665; doi:10.1002/advs.201600137)
Supplement: Supplementary file 1 — Supplementary [file ADVS-3-0i-s001.pdf]

Copyright WILEY-VCH Verlag GmbH & Co. KGaA, 69469 Weinheim, Germany, 2016.

ADVANCED SCIENCE Open Access

Supporting Information

for *Adv. Sci.,* DOI: 10.1002/advs.201600137

Periodic Organic–Inorganic Halide Perovskite Microplatelet Arrays on Silicon Substrates for Room-Temperature Lasing

Xinfeng Liu, Lin Niu, Chunyang Wu, Chunxiao Cong, Hong Wang, Qingsheng Zeng, Haiyong He, Qundong Fu, Wei Fu, Ting Yu, Chuanhong Jin, Zheng Liu,* and Tze Chien Sum**

Supporting Information

Periodic Organic–Inorganic Halide PerovskiteMicro-platelet Arrays on Silicon Substratesfor Room-Temperature Lasing

XinfengLiu,[1,2,†,] Lin Niu,[3,†] Chunyang Wu,[4] Chunxiao Cong,[2] Hong Wang,[3] Qingsheng Zeng,[3] Haiyong He,[3] Qundong Fu,[3] Wei Fu,[3] Ting Yu,[2] Chuanhong Jin,[4] Zheng Liu,[3,5,*] Tze Chien Sum[2,6,*]*

[1]CAS Center for Excellence in Nanoscience & CAS Key Laboratory of Standardization and Measurement for Nanotechnology, National Center for Nanoscience and Technology, Beijing 100190, China

[2]Division of Physics and Applied Physics, School of Physical and Mathematical Sciences, Nanyang Technological University, Singapore 637371

[3]Center for Programmable Materials, School of Materials Science & Engineering, Nanyang Technological University, Singapore 639798, Singapore

[4]State Key Laboratory of Silicon Materials, School of Materials Science and Engineering, Zhejiang University, Hangzhou 310027, China

[5]NOVITAS, Nanoelectronics Centre of Excellence, School of Electrical and Electronic Engineering, Nanyang Technological University, 639798, Singapore

[6]Energy Research Institute @ NTU (ERI@N), Nanyang Technological University, 50 Nanyang Drive, Singapore 637553

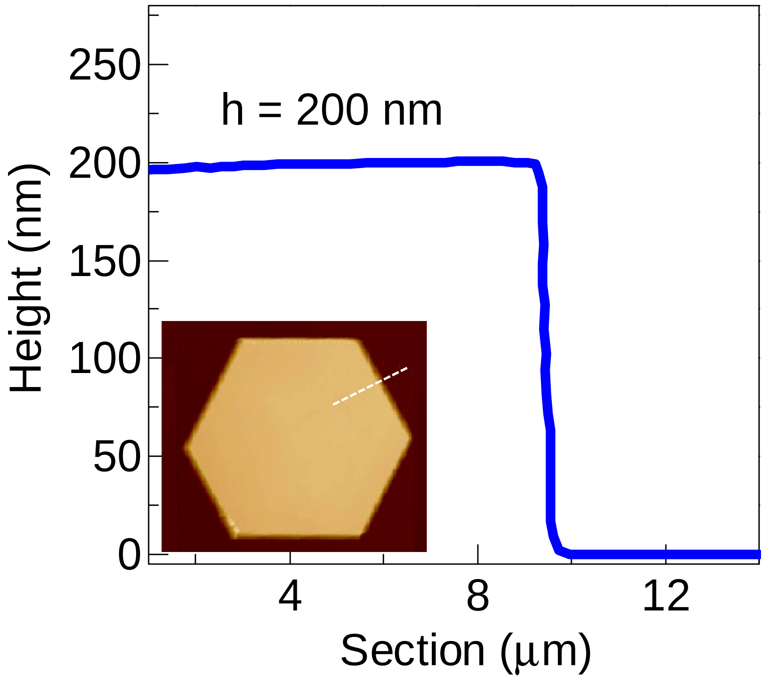

Figure S1 AFM measurement of a perovskite platelet. AFM image of a perovskite platelet with an edge of ~15 μm; the blue line is the profile of the dashed white line. The thickness of the perovskite is ~200 nm. The AFM image shows a very flat surface, which is excellent for lasing in such hexagonal cavity.

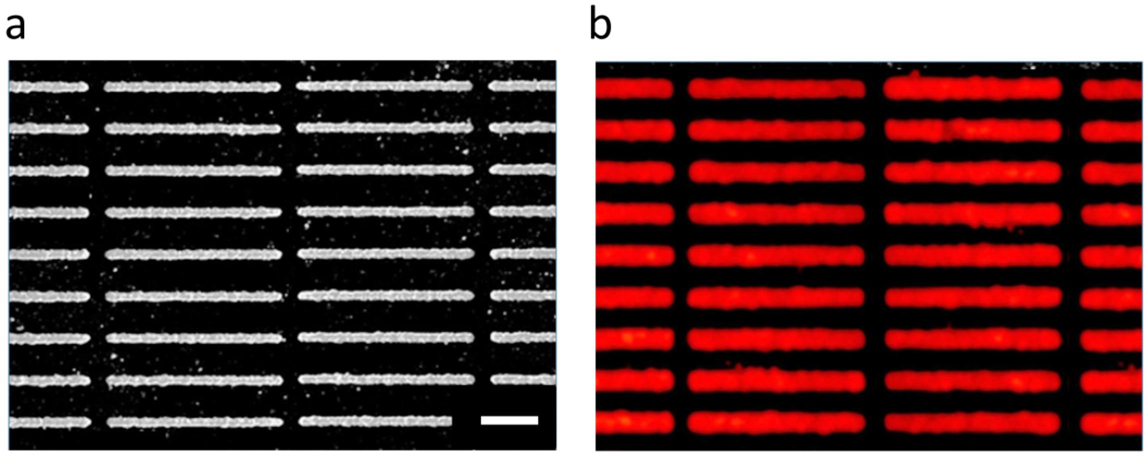

Figure S2 SEM and PL characterization of fabricated perovskite nanowire array. (a) The SEM image of as-fabricated perovskite nanowire array. The scale bar is 5 μm. (b) PL image of as-fabricated perovskite nanowire array. The excitation wavelength is 400 nm and the pump laser is filtered by a 500 nm long-pass filter. The PL image was collected using the emission peak at around 780 nm.

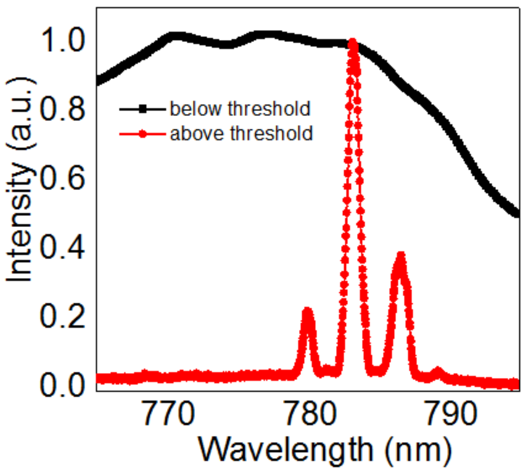

Figure S3 The emission spectra from perovskite sample when the pump fluence is below (black dots) and above threshold (red dots).

Figure S3 show the narrowing of the emission peak when the pump fluence is above the threshold. It is clear that when the lasing occurs in the perovksite cavity, very sharp peaks can be observed with the narrowing of the FWHM.

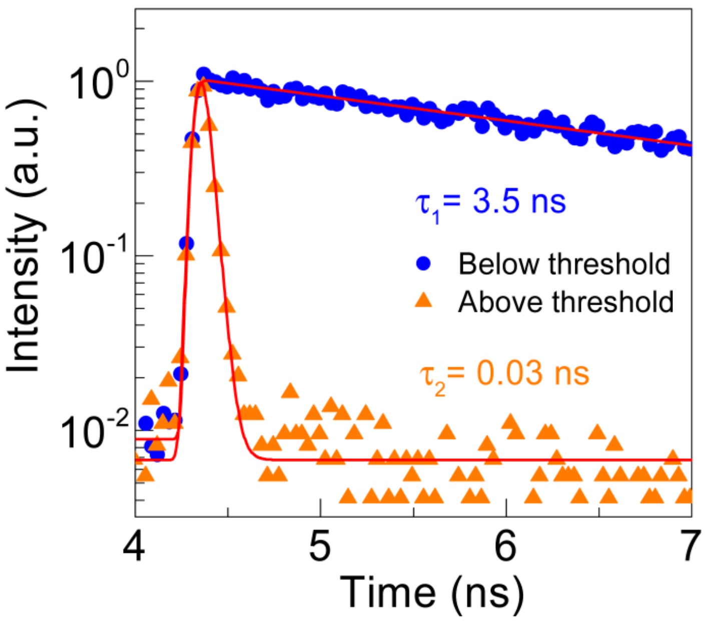

Figure S4 Lifetime measurement of perovskite platelet with pump fluence below and above threshold. The PL wavelength is at 780 nm, which corresponds to the band edge emission of the perovskite. When the pump fluence is below the threshold (~5 μJ/cm^2), the lifetime of perovskite emission is 3.5 ns, which is consistent with previous report; when the pump fluence is high enough (~40 μJ/cm^2), only one fast lifetime (~30 ps – that is limited by the streak camera resolution over this few ns time window) can be observed. The very fast lifetime is an obvious feature of the occurrence of lasing.

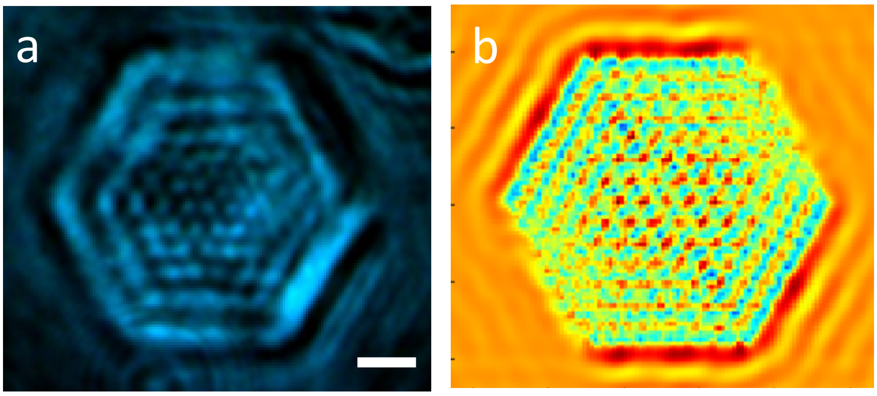

Figure S5 Scattering pattern of excitation laser and the corresponding simulation result. (a) The optical image of scattering pattern at the surface of perovskite platelet. The formation of this pattern is ascribed to the excellent confinement afforded by the hexagonal cavity. (b) The electrical field distribution at excitation wavelength is simulated here; the similar pattern shape suggests the good confinement in the cavity. The scale bar is 5 μm.

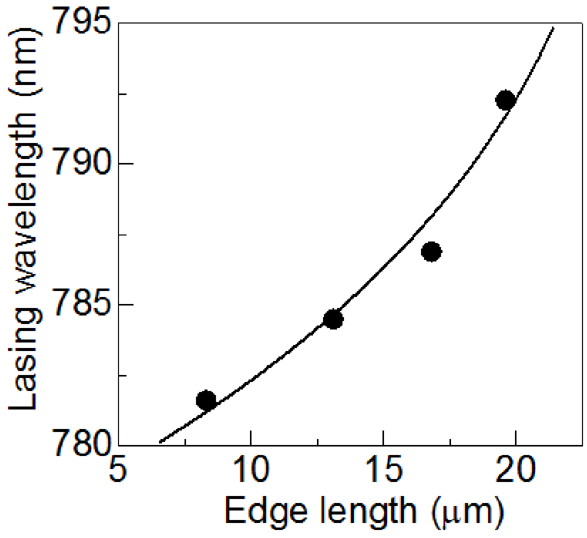

Figure S6 Edge length dependent lasing wavelength for different sized perovskite platelets. The wavelength of lasing mode is plotted as a function of the hexagonal cavity edge length. The relationship can be well-fitted by a single-exponential increasing function.

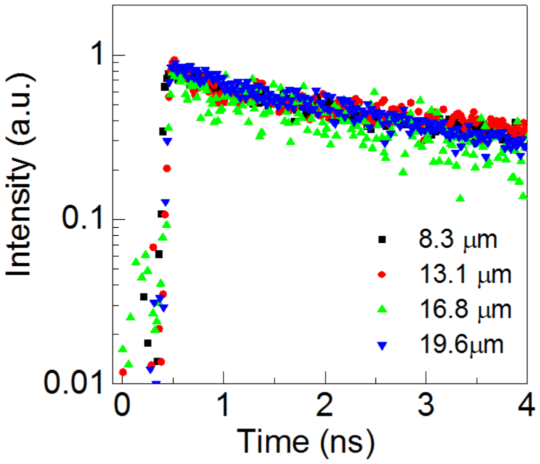

Figure S7 Time-resolved photoluminescence decay profile of perovskite platelet with different size. From the decay profile, it is evident that these samples share similar decay lifetimes for different perovskite platelet size.

References:

[1] Casperson, Lee W., Journal of Applied Physics, 46, 5194-520 (1975).
[2] Zimmler, Mariano A.; Jiming Bao; Federico Capasso; Sven Müller; Carsten Ronning, Applied Physics Letters, 93, 051101 (2008)
